# Supplementary material for: Free-space-coupled wavelength-scale disk resonators
Source: Nanophotonics. 2022 Apr 20;11(12):2901–8. doi: 10.1515/nanoph-2022-0106 (PMC11501722; doi:10.1515/nanoph-2022-0106)
Supplement: Supplementary file 1 — Supplementary Material [file j_nanoph-2022-0106_suppl.docx]

***Supplementary Information for***

**Free-space-coupled wavelength-scale disk resonators**

Babak Mirzapourbeinekalaye, Sarath Samudrala, Mahdad Mansouree, Andrew McClung, and Amir Arbabi

Department of Electrical and Computer Engineering, University of Massachusetts Amherst, 151 Holdsworth Way, Amherst, MA 01003, USA


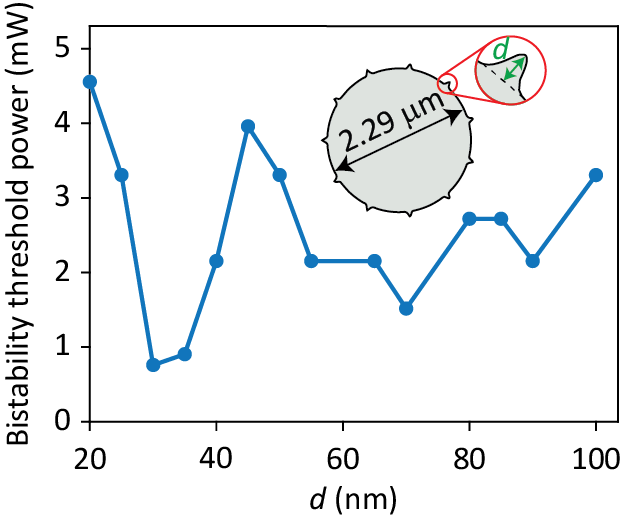


**Figure S1.** Measured minimum incident power for observing optical bistability for a set of resonators with different grating protrusion depth $d$. The boundary of the microdisk is defined in the polar coordinates according to $\rho=R_{0}+d{(\sin m\phi)}^{n}$, where $R_{0}=1.145$ µm, $m=5.5$, and $n=100$.

**Supplementary Note 1: Temporal coupled-mode model for free-space-coupled resonators**

Here we present a universal model for the excitation of resonators by freely propagating waves. The model describes the dynamic response of the resonator and can be used to determine the stored energy and the power absorbed in the resonator. Consider the resonator shown in Fig. S2a that is excited by a narrowband incident wave. For simplicity, we assume the electric field of the resonant mode $\mathbf{E}^{M}$ is normalized such that it radiates unit power. The phasor of the electric field of the resonant mode in the far-field can be written as

| $\mathbf{E}_{\mathrm{ff}}=\frac{-jk_{0}\eta_{0}}{\pi r}e^{-jk_{0}r}\mathbf{F}\left( \theta,\phi\right)$, | (S1) |
| --- | --- |

where $k_{0}=\frac{\omega_{0}}{c}$ is the wavenumber at the resonant frequency $\omega_{0}$, $c$ is the speed of light in a vacuum, $\eta_{0}$ is the free-space impedance, and $\mathbf{F}(\theta, \phi)$ specifies the radiation pattern of the resonator. The directivity of the resonant mode pattern is given by^1^

| $D\left( \theta,\phi\right)=\frac{2{\eta_{0}k}_{0}^{2}}{\pi}\left\vert\mathbf{F}\left( \theta,\phi\right) \right\vert^{2}$. | (S2) |
| --- | --- |


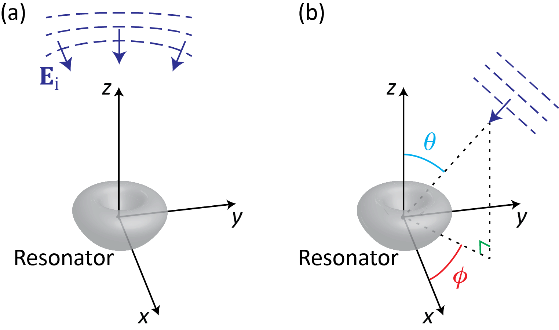


**Figure S2.** (a) Schematic illustration of a resonator excited by an incident wave. (b) Schematic illustration of a resonator excited by a plane wave incident from spherical coordinates direction $(\theta,\phi)$.

The dynamics of a driven resonator can be described using the time-domain coupled-mode model that describes the time evolution of the energy amplitude of the resonant mode^2^. The positive-frequency component of the electric field of the incident wave can be expressed as

| $\mathbf{E}_{i}\mathcal{=E}\left( t \right)e^{j\omega t}{\tilde{\mathbf{E}}}_{i}(\mathbf{r})$, | (S3) |
| --- | --- |

where $\mathcal{E(}t)$ is a slow varying complex-valued excitation amplitude, $\omega$ is the center frequency, and ${\tilde{\mathbf{E}}}_{i}(\mathbf{r})$ defines the spatial distribution of the incident wave.

The evolution of the energy amplitude of the resonant mode is described by

| $\frac{da}{dt}=j\omega_{0}a-\frac{1}{2\tau}a+\kappa\mathcal{E}\left( t \right)e^{j\omega t}$, | (S4) |
| --- | --- |

where $a$ is the energy amplitude of the resonant mode (i.e., the stored energy of the resonant mode is given by $U_{s}=\left| a \right|^{2}$), $\omega_{0}$ is the resonant frequency, $\kappa$ is the coupling coefficient representing the coupling strength between the incident wave and the resonant mode, and $\tau=Q/\omega_{0}$ is the photon lifetime^2^. The photon loss rate is given by $1/\tau$, has contributions from the radiation and absorption losses, and can be written as $1/\tau=1/\tau_{r}+1/\tau_{a}$ where $\tau_{r}$ and $\tau_{a}$ are photon radiation and absorption lifetimes.

**Coupling coefficient for plane wave excitation**

We first find an expression for the coupling coefficient $\kappa$ for plane wave excitation and then determine $\kappa$ for more general excitations by expanding them in terms of plane waves. We assume that the resonator is excited by a narrowband plane wave that is incident from the spherical coordinates direction $(\theta,\phi)$, as shown in Fig. S2b. The positive-frequency component of the electric field of the incident wave can be expressed as

| $\mathbf{E}_{i}\mathcal{=E}\left( t \right)e^{j\omega t}e^{-j\mathbf{k}_{i}\cdot\mathbf{r}}\hat{e}$, | (S5) |
| --- | --- |

where $\mathbf{k}_{i}=-k_{0}(\sin\theta\cos\phi,\sin\theta\sin\phi,\cos\theta)$ is the wave vector, $k_{0}=\frac{\omega}{c}$, and $\hat{e}$ is a unit vector defining the polarization. To find an expression for $\kappa$, we consider the special case when the resonator has no absorption lossless (i.e., $\tau=\tau_{r}$) and the incident wave is monochromatic with frequency $\omega_{0}$, that is $\mathcal{E}\left( t \right)e^{j\omega t}=E_{0}e^{j\omega_{0}t}$ where $E_{0}$ is a constant. Note that $\kappa$ is independent of absorption losses and $\mathcal{E}\left( t \right)$, and these assumption does not affect its value. Under such an excitation, $a=\tilde{a}e^{j\omega_{0}t}$ and $\tilde{a}$ is found from (S4) as

| $\tilde{a}=2\tau\kappa E_{0}$. | (S6) |
| --- | --- |

For a resonator with no absorption loss, the excitation amplitude of the resonant mode (the amplitude of $\mathbf{E}^{M}$ in the modal expansion of the scattered field) is given by^3,4^

| $b=\frac{1}{2}\int\mathbf{E}_{i}\cdot\mathbf{J}^{M}dv$, | (S7) |
| --- | --- |

where $\mathbf{J}^{M}$ is the equivalent polarization current density of the mode ($\mathbf{J}^{M}=j\omega_{0}\epsilon_{0}\left( \epsilon_{r}-1 \right)\mathbf{E}^{M}$). For a plane wave incident, the right hand of (S7) is a Fourier transform and is related to the radiation pattern of the current (which is the same as the mode) by^1^

| $b=2E_{0}\mathbf{F}\left( \theta,\phi\right)\cdot\hat{e}$. | (S8) |
| --- | --- |

Since we assumed $\mathbf{E}^{M}$ is power normalized, the power radiated by the excited resonant mode (i.e., $b\mathbf{E}^{M}$) is $\left| b \right|^{2}$. The radiated power is related to the stored energy of the mode $\left| \tilde{a} \right|^{2}$ by the radiative photon lifetime $(P_{\mathrm{rad}}=U_{s}/\tau_{r})$, thus

| $b=\tilde{a}\sqrt{1/\tau_{r}}$. | (S9) |
| --- | --- |

From Eqs. (S6), (S8), and (S9), we find

| $\tilde{a}=2\sqrt{\tau_{r}}E_{0}\mathbf{F}\left( \theta,\phi\right)\cdot\hat{e}={2\tau}_{r}\kappa E_{0}$, | (S10) |
| --- | --- |

thus

| $\kappa=\sqrt{\frac{1}{\tau_{r}}}\mathbf{F}\left( \theta,\phi\right)\cdot\hat{e}$. | (S11) |
| --- | --- |

**Coupling coefficient for general excitation**

A more general coherent incident wave can be expanded in terms of plane waves, and the positive-frequency component of the electric field of the incident wave can be expressed as

| $\mathbf{E}_{i}\mathcal{=E}\left( t \right)e^{j\omega t}{\tilde{\mathbf{E}}}_{i}(\mathbf{r}\mathcal{)=E}\left( t \right)e^{j\omega t}\int\boldsymbol{e}\left( \theta,\phi\right)e^{-j\mathbf{k}_{i}\cdot\mathbf{r}}d\Omega$, | (S12) |
| --- | --- |

where $d\Omega=\sin\theta d\theta d\phi$is the solid angle differential, and $\boldsymbol{e}(\theta,\phi)$ is the vector amplitude of the plane wave in the expansion of ${\tilde{\mathbf{E}}}_{i}(\mathbf{r})$ that is incident from the spherical coordinates’ direction $(\theta,\phi)$. $\boldsymbol{e}(\theta,\phi)$ specifies the far-field radiation pattern of the incident wave. When $\mathbf{E}_{i}$ is incident from the $z>0$ half-space, $\boldsymbol{e}$ is given by

| $\boldsymbol{e}(\theta,\phi)=k^{2}\cos\theta\mathbb{E}_{i}(-k_{0}\sin\theta\cos\phi, -k_{0}\sin\theta\sin\phi)$, | (S13) |
| --- | --- |

where $\mathbb{E}_{i}(k_{x},k_{y})$ is the spatial Fourier transform of ${\tilde{\mathbf{E}}}_{i}(\mathbf{r})$ in the $z=0$ plane

| $\mathbb{E}_{i}\left( k_{x},k_{y} \right)=\frac{1}{4\pi^{2}}\int\int{\tilde{\mathbf{E}}}_{i}\left( x,y,z=0 \right)e^{j\left( k_{x}x\boldsymbol{+}k_{y}y \right)}dxdy$. | (S14) |
| --- | --- |

The coupling coefficient for the general excitation is the sum of the coupling coefficients for different plane waves and is given by

| $\kappa=\sqrt{\frac{1}{\tau_{r}}}\int\mathbf{F}\left( \theta,\phi\right)\cdot\boldsymbol{e}\left( \theta,\phi\right)d\Omega$. | (S15) |
| --- | --- |

The time-averaged power of the incident wave is given by

| $P_{\mathrm{in}}=\left\vert\mathcal{E}\left( t \right) \right\vert^{2}\frac{2\pi^{2}}{\eta_{0}k^{2}}\int\left\vert\boldsymbol{e} \right\vert^{2}d\Omega$. | (S16) |
| --- | --- |

Based on (S15) and (S16) and according to the Cauchy–Schwarz inequality $\kappa$ is maximized when $\boldsymbol{e}(\theta,\phi)$ is proportional to $\mathbf{F}^{\mathbf{*}}\left( \theta,\phi\right)$ and its maximum value is $\kappa_{\max} =\sqrt{\frac{1}{\tau_{r}}}\frac{\sqrt{P_{\mathrm{in}}}}{\left| \mathcal{E}\left( t \right) \right|}$. For a given incident power, the maximum stored energy is achieved when $\omega=\omega_{0}$, there is no absorption loss, and $\kappa$ has its maximum value. The maximum stored energy is given by $U_{s_{\max}}=4\tau_{r}P_{\mathrm{in}}$. To increase coupling to the resonator, the overlap integral in (S15) should be maximized. For example, when exciting the resonator by a focused beam, the numerical aperture of the focusing lens should be selected such that the convergence of the incident beam matches the divergence of the radiation pattern of the resonator.

**Supplementary Note 2: Stored energy, reradiated and absorbed powers**

Here we find the time-average stored energy, reradiated power, and absorbed power when the resonator is excited by a plane wave at its resonant frequency $\omega_{0}$. Plugging in $\kappa$ from (S11) in (S6), the phasor of the energy amplitude is found as

| $\tilde{a}=2\tau\sqrt{\frac{1}{\tau_{r}}}\mathbf{F}\left( \theta,\phi\right)\cdot\hat{e}E_{0}$. | (S17) |
| --- | --- |

The stored energy

| $U_{s}=\left\vert\tilde{a} \right\vert^{2}=\frac{4\pi\tau^{2}}{k_{0}^{2}\tau_{r}}D\frac{1}{2\eta_{0}}\left\vert E_{0} \right\vert^{2}=\frac{Q^{2}D}{2\pi^{2}Q_{r}}\left( \frac{1}{2}\epsilon_{0}\left\vert E_{0} \right\vert^{2}\lambda_{0}^{3} \right)=\frac{u_{i}\lambda_{0}^{3}}{2\pi^{2}}\frac{Q^{2}D}{Q_{r}}$, | (S18) |
| --- | --- |

where we have used (S2) and have represented $D(\theta, \phi)$ by $D$ for brevity, and $u_{i}=\frac{1}{2}\epsilon_{0}\left| E_{0} \right|^{2}$ is the energy density of the incident wave. For a resonator with no absorption loss $Q=Q_{r}$ and (S18) reduces to (1). Figure S3 shows the normalized stored energy $U_{s}/(u_{i}\lambda_{0}^{3})$ for the resonator shown in Fig. 1d computed using (1) and using direct integration of energy density obtained by full-wave simulations inside the resonator volume. The stored energy obtained using full-wave simulation contains small contributions from other non-resonant modes and is expected to be slightly larger.

**
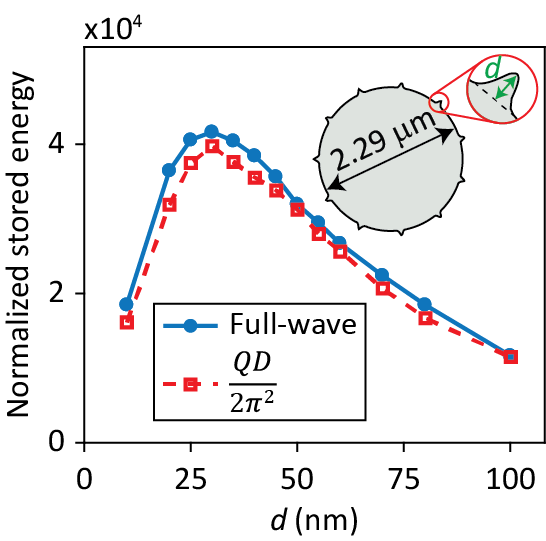
**

**Figure S3.** Normalized stored energy $U_{s}/(u_{i}\lambda_{0}^{3})$ as a function of grating protrusion $d$ obtained using full-wave simulations and using (1). A schematic of the resonator used in the simulations is shown in the inset. The boundary of the microdisk is defined in the polar coordinates according to $\rho=R_{0}+d{(\sin m\phi)}^{n}$, where $R_{0}=1.145$ µm, $m=5.5$, and $n=100$.

The resonator scatters some of the incident power, and a portion of the scattered power can be considered as the reradiation by the excited resonant mode. The reradiated power of the mode is given by

| $P_{\mathrm{rr}}^{M}=\frac{U_{s}}{\tau_{r}}=4\pi\frac{I_{0}}{k_{0}^{2}}\left( \frac{\tau}{\tau_{r}} \right)^{2}D=4A_{\mathrm{eff}}I_{0}\left( \frac{\tau}{\tau_{r}} \right)^{2}$, | (S19) |
| --- | --- |

where $I_{0}=\frac{1}{2\eta_{0}}\left| E_{0} \right|^{2}$ is the intensity of the incident plane wave, and $A_{\mathrm{eff}}=\frac{D\lambda_{0}^{2}}{4\pi}$ is the effective area of the resonator, which is the same expression used for the effective area of an antenna^1^. The scattering cross-section *due to the resonant mode* of the resonator is

| $\sigma_{\mathrm{scat}}^{M}=4A_{\mathrm{eff}}\left( \frac{\tau}{\tau_{r}} \right)^{2}$. | (S20) |
| --- | --- |

$\sigma_{\mathrm{scat}}^{M}$ is maximum for a resonator with no absorption loss and is equal to $4A_{\mathrm{eff}}$. Note that the optical power scattered by the resonant mode might not be a significant portion of the total scattered power, and the total scattering cross-section of a high-$Q$ resonator might be significantly larger than $\sigma_{\mathrm{scat}}^{M}$.

The power absorbed by the excited resonant mode is given by

| $P_{a}=\frac{U_{s}}{\tau_{a}}=U_{s}\left( \frac{1}{\tau}-\frac{1}{\tau_{r}} \right)=4A_{\mathrm{eff}}I_{0}\frac{\tau}{\tau_{r}}(1-\frac{\tau}{\tau_{r}})$, | (S21) |
| --- | --- |

where we have used (S19) for $U_{s}$. For high-$Q$ resonators whose $A_{\mathrm{eff}}$ are not very small, the power absorbed by the resonant mode is the dominant portion of total absorbed power, and the absorption cross-section of the resonator is given by

| $\sigma_{\mathrm{abs}}=4A_{\mathrm{eff}}\frac{\tau}{\tau_{r}}(1-\frac{\tau}{\tau_{r}})$. | (S22) |
| --- | --- |

The absorption cross-section is maximum when $\tau=1/\tau_{r}$ or $\tau_{r}=\tau_{a}$ and its maximum value is $A_{\mathrm{eff}}.$ Therefore, the maximum power is absorbed by a resonator when its radiative and absorptive losses are equal, and we refer to such a resonator as a matched resonator. The maximum power that a resonator can absorb is

| $P_{a_{\max}}=A_{\mathrm{eff}}I_{0}$, | (S23) |
| --- | --- |

which is the same as the maximum power that can be received by an impedance-matched antenna^1^.

**References**

1. Balanis, C. A. *Antenna theory: analysis and design*. (John Wiley & Sons, 2015).

2. Haus, H. *Waves and Fields in Optoelectronics*. *Prentice-Hall* (Prentice-Hall, 1985).

3. Harrington, R. & Mautz, J. Theory of characteristic modes for conducting bodies. *IEEE Trans. Antennas Propag.* **19**, 622–628 (1971).

4. Harrington, R., Mautz, J. & Yu Chang. Characteristic modes for dielectric and magnetic bodies. *IEEE Trans. Antennas Propag.* **20**, 194–198 (1972).


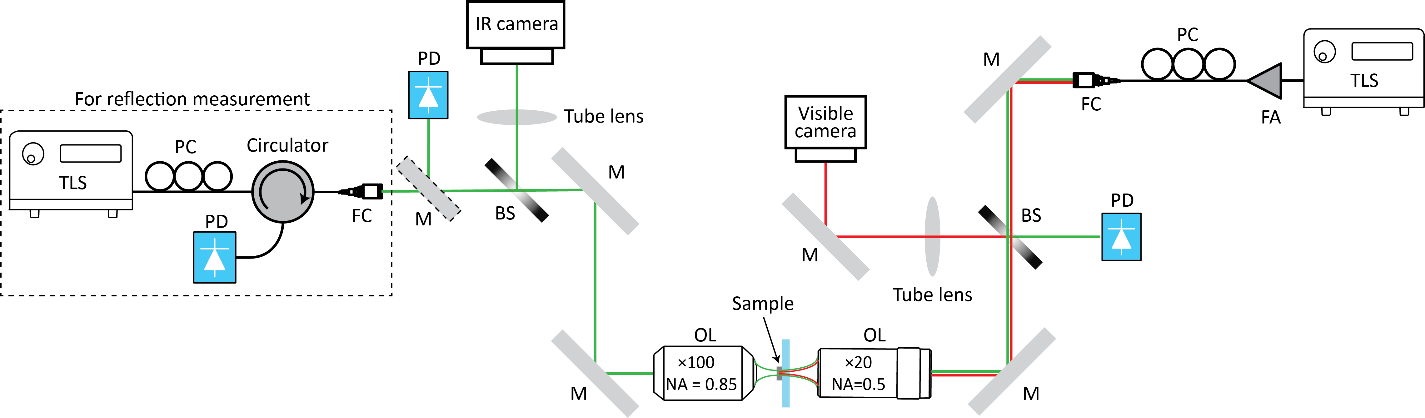


**Figure S4.** Schematic illustration of the characterization setup used for measuring transmission and reflection spectra and of microdisks at different power levels. PD: photodetector, M: mirror, BS: beam splitter, OL: objective lens, PC: polarization controller, FA: fiber amplifier FC: fiber collimator. The red and green lines depict the visible and infrared beam paths, respectively. The visible laser and camera are used for the imaging and alignment of the sample.

**
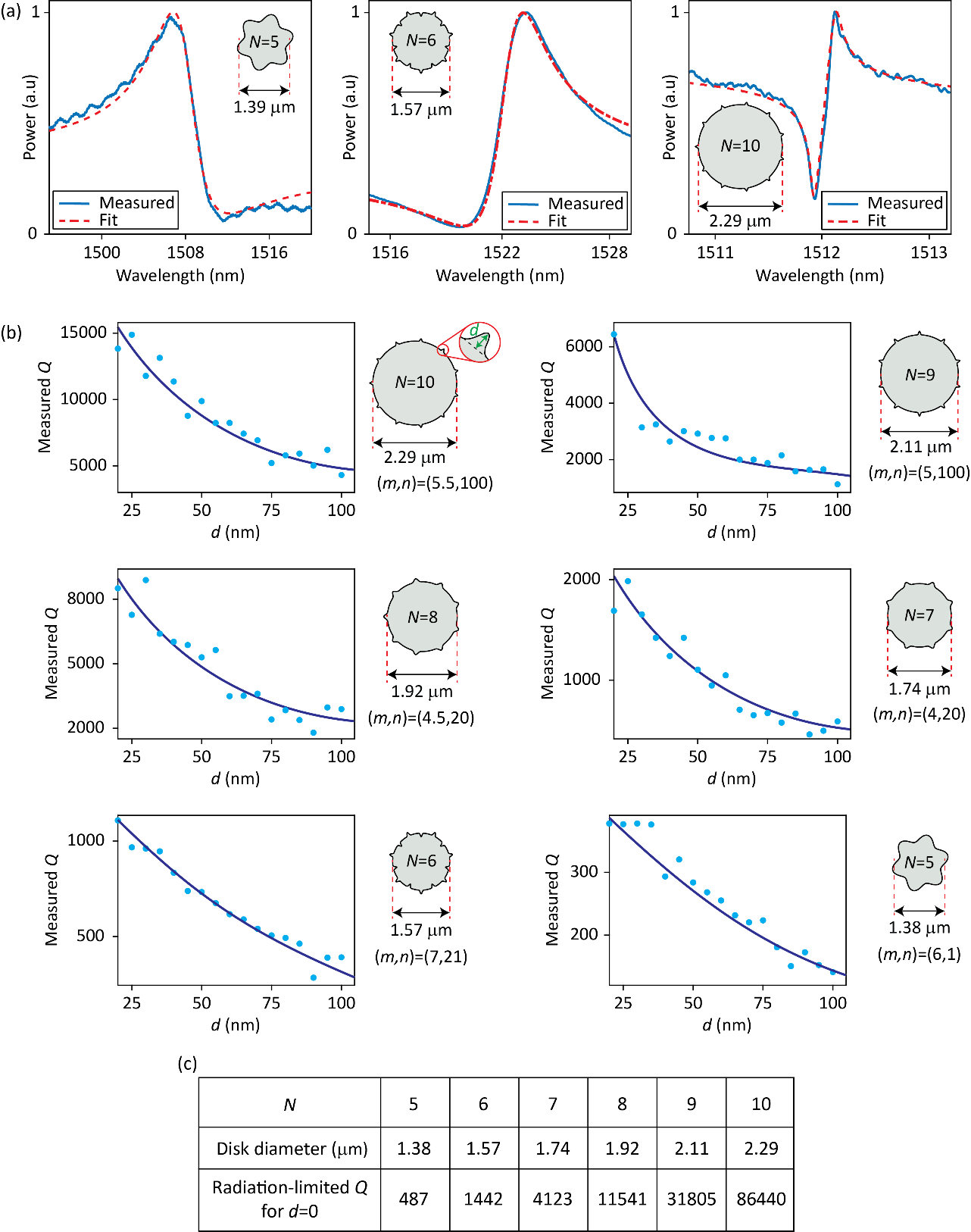
**

**Figure S5.** (a) Measured transmission spectra for three FSC microdisks representing Fano lineshapes. The boundary of the microdisk is defined in the polar coordinates according to $\rho=R_{0}+d{(\sin m\phi)}^{n}$, where $R_{0}$ is the microdisk radius. The microdisks and their diameters are shown in insets, and $\left( d, m, n \right)$ are (80 nm, 6, 1), (40 nm, 7, 21), and (35 nm, 5.5, 100) for the three devices from left to right, respectively. Dashed lines are Fano lineshape fits. (b) Measured quality factors of an array of devices with different designs as functions of grating strengths $d$. (c) Simulated radiation-limited quality factors of the resonators presented in (b) with no protrusion ($d=0$).
